# Day to Day Variability and Effects of Collection Conditions on Urinary Renin‐Angiotensin‐Aldosterone System Biomarkers in Vervet Monkeys (*Chlorocebus aethiops sabaeus*)

**DOI:** 10.1111/jmp.70131

**Published:** 2026-11-11

**Authors:** Mary Elaine Graham, Kelli Carter, Tanya M. Gwathmey, Sarah N. Cilvik

**Affiliations:** ^1^ Department of Pediatrics Wake Forest University School of Medicine Winston‐Salem North Carolina USA; ^2^ Department of Surgery Wake Forest University School of Medicine Winston‐Salem North Carolina USA; ^3^ Biomarker Analytical Core Atrium Health/Wake Forest University School of Medicine Winston‐Salem North Carolina USA

**Keywords:** African green monkey, cystocentesis, non‐human primate, RAAS, urinalysis

## Abstract

**Background:**

Dysregulation of the renin‐angiotensin‐aldosterone system (RAAS) is associated with cardiovascular and renal disease. Our objectives were to determine the daily variability in urinary RAAS biomarkers and differences between non‐sedated free catch (FC) and sedated cystocentesis samples in vervet monkeys.

**Methods:**

We performed repeated urine collections (six non‐sedated FC, three sedated cystocentesis) from six adult vervets (*Chlorocebus aethiops sabaeus*). We quantified RAAS components, normalized to urine creatinine. We assessed variability using coefficients of variation (CV) and compared sedation effects using linear mixed‐effects models with false discovery rate correction.

**Results:**

Analytes showed high to very high variability (CV 28.17%–103.03%). Angiotensin converting enzyme demonstrated the lowest variability (cystocentesis, CV 28.17% ± 18.28%). Creatinine, Angiotensin‐(1–7), Angiotensin II, and aldosterone concentrations differed between non‐sedated FC and sedated cystocentesis samples.

**Conclusions:**

Substantial within‐animal variability suggests that single‐timepoint measurements insufficiently characterize urinary RAAS in vervets. Differences between collection conditions support standardization of collection methods and repeated sampling.

## 1 Introduction

The renin‐angiotensin‐aldosterone system (RAAS), comprised of angiotensin converting enzymes (ACE, ACE2), angiotensin peptides [Ang‐(1–7), Ang II], and aldosterone, plays a major role in blood pressure regulation, fluid balance, kidney function, and cardiovascular health [1]. Dysregulation of this system has been associated with excessive vasoconstriction, inflammation, fibrosis, and development of cardiovascular and renal disease [1, 2, 3, 4].

Standard RAAS analysis relies on the measurement of circulating biomarkers in plasma or serum, including renin, aldosterone, and angiotensin II, which reflect renal renin release, hepatic angiotensinogen production, pulmonary ACE activity, and adrenal aldosterone secretion [5]. These biomarkers are regulated by a negative feedback loop, respond within minutes to acute stimuli, and have short half‐lives (e.g., approximately 15 min for aldosterone and renin) [6, 7]. Measurement is further complicated by preanalytical factors (e.g., posture, medications, electrolyte status, hydration), by collection and processing protocols that are stringent and difficult to implement (e.g., timed, temperature‐controlled sample handling), and by limitations of laboratory assays [8]. RAAS biomarkers also vary within individuals over time, such as across the circadian and menstrual cycles [9]. As a result, samples represent snapshots in time of circulating RAAS activity that may not reflect baseline activity [9]. Despite these limitations, careful collection of serum and plasma biomarkers with analysis in specialized laboratories and interpretation by experts enables measurement of systemic RAAS activity in humans [7].

In nonhuman primate (NHP) models, measurement of serum RAAS activity is subject to similar variability and is compounded by the need for venipuncture under sedation due to the relatively large blood volumes required for assays. Because sedation impacts cardiovascular function, sympathetic activity, and blood pressure, sampling under sedation may itself influence RAAS measurements. Existing studies have demonstrated impacts of sedatives on blood pressure, heart rate, cardiac output, and ventricular function, suggesting that samples obtained during sedation may not reliably reflect baseline activity [10, 11, 12, 13].

Measurement of RAAS biomarkers and related factors (e.g., alpha‐klotho) in urine offers a potentially accessible means of evaluating pathway activity without sedation and without the invasive sampling procedures currently required to assess RAAS function in NHP models [14, 15, 16]. However, accurate interpretation of urinary biomarker concentrations requires an understanding of both biologic variability and methodological factors that may influence measurement. Day‐to‐day fluctuations in biomarker concentrations can arise from physiologic changes in hydration status, diet, circadian rhythms, and hormonal regulation [17]. Urine collection technique may introduce further variability. In laboratory animal research, urine is commonly collected either by free‐catch (FC) sampling or by cystocentesis. While FC collection is noninvasive and can be performed without sedation, cystocentesis provides a more controlled sample and may reduce contamination from the lower urinary tract [15]. However, sedation required for cystocentesis may alter RAAS activity [11, 12, 18, 19, 20, 21, 22, 23, 24], potentially affecting urinary biomarker measurements. These effects are poorly characterized, particularly in NHP urine samples.

Despite widespread use of urinary biomarkers in animal studies, limited data are available regarding the variability of urinary RAAS biomarkers or the impact of sedation and urine collection method on measured concentrations in NHP. Understanding these sources of variability is essential for study design, interpretation of longitudinal data, and comparison of findings across studies. Therefore, the objectives of this study were to characterize day‐to‐day variability of urinary RAAS biomarkers and related analytes in vervet monkeys and to evaluate differences in biomarker concentrations between non‐sedated FC and sedated cystocentesis urine samples. Given physiological variability, sedation‐related effects on RAAS activity, and differences in FC versus cystocentesis collection method, we hypothesized that urinary RAAS biomarker concentrations would demonstrate significant day‐to‐day variability and that biomarker concentrations would differ between non‐sedated FC and sedated cystocentesis urine samples.

## 2 Materials and Methods

### 2.1 Humane Care Guidelines

The authors confirm adherence to the ethical policies of the journal, as noted on the journal's author guidelines page. We performed all animal studies at Wake Forest University School of Medicine (WFUSM), which is accredited by the Association for the Assessment and Accreditation of Laboratory Animal Care (AAALAC). We conducted studies following protocol approval by the WFUSM Institutional Animal Care and Use Committee (protocol number A24‐016). All animal procedures conformed to the requirements of the USDA Animal Welfare Act and complied with international, national, and institutional guidelines for humane animal treatment, including those set forth by the US National Research Council and the US Public Health Service “Policy on Humane Care and Use of Laboratory Animals” and “Guide for the Care and Use of Laboratory Animals.”

### 2.2 Subject Selection, Animal Care, and Sample Collection

We selected three male and three non‐pregnant female historically healthy (non‐hypertensive, non‐diabetic, and non‐obese) adult vervet monkeys (*Chlorocebus aethiops sabaeus*) between the ages of 7 and 9 years old from the Vervet Research Colony (VRC) at WFUSM (Table 1, Figure 1). This colony is a multigenerational pedigreed, genomically‐sequenced, socially housed breeding colony [25]. These animals were selected as part of a larger study (not yet published) investigating developmental programming of cardiovascular health using echocardiography, cardiac magnetic resonance imaging, and cardiovascular biomarker analysis. Sampling days were determined by the imaging schedule for that study.

**TABLE 1:** Demographics and sample collection.

Demographics	
Number of vervets	6
Sex (M/F)	3/3
Age, years (mean ± SD)	7.61 ± 0.78
Weight, kg (mean ± SD)	6.24 ± 2.09

Abbreviations: F, female; M, male; SD, standard deviation.

**FIGURE 1:**
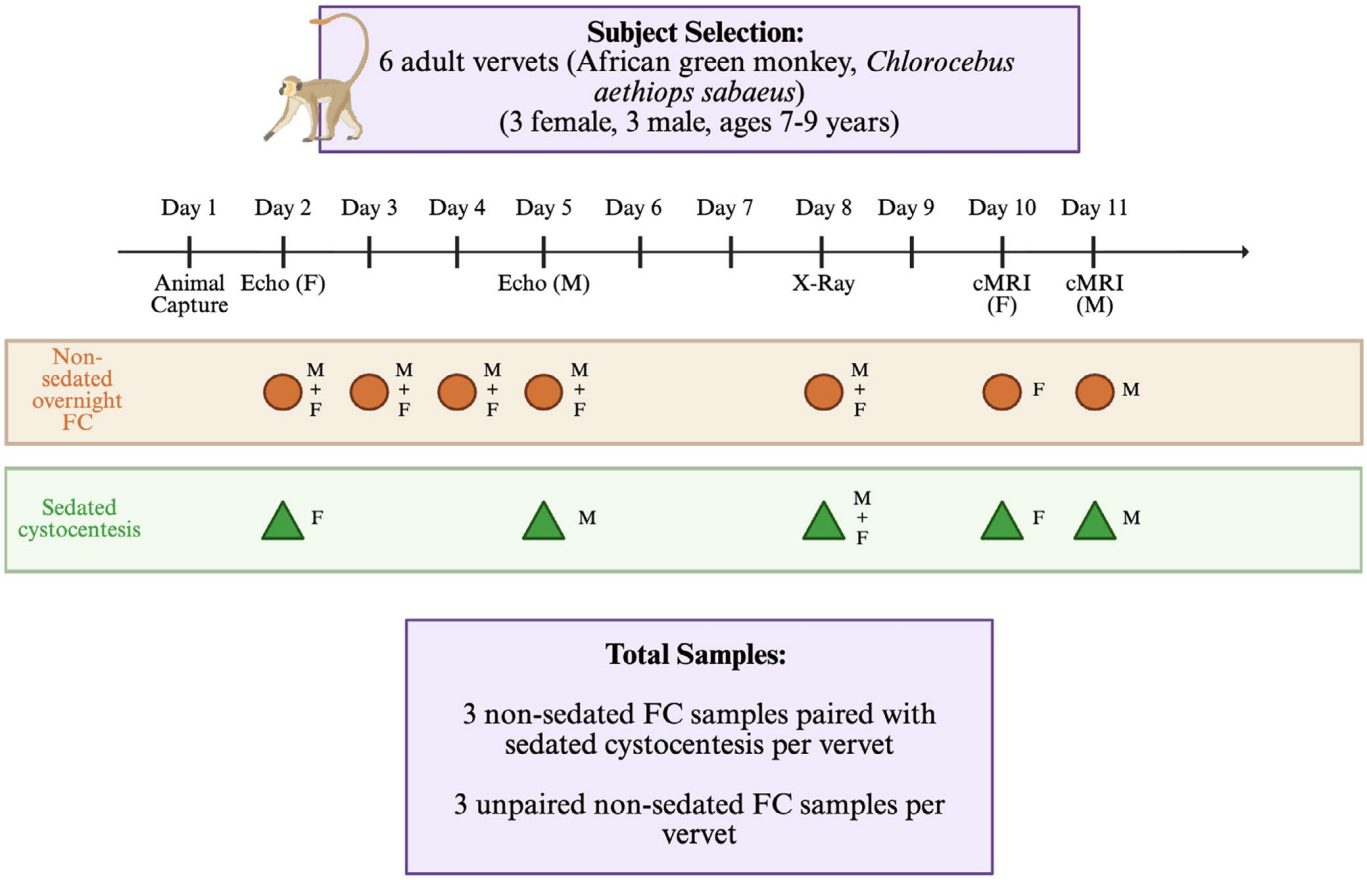
Sample collection timeline. Six total non‐sedated FC and three total sedated cystocentesis samples were collected per vervet, with exceptions and exclusions detailed in methods. cMRI, cardiac magnetic resonance imaging; echo, echocardiography; F, female; FC, free catch; M, male. Created in BioRender. Graham, M. (2026) https://BioRender.com/6uy9lk1. The publication license (agreement number GI2A5F1YGV) was obtained 9/1/2026.

VRC staff temporarily removed selected animals from their normal housing (large indoor‐outdoor social groups) and placed them in rack housing on study day 1, where each animal was housed in its own rack compartment with physical, visual, and auditory contact with neighboring animals. Each compartment had its own pan, so FC urine could be attributed to individual monkeys. All animals had ad libitum access to food (commercial standard laboratory monkey chow, Lab Diet 5038; Purina, St. Louis, MO) and water. We removed food access at least 12 h prior to sedated studies; monkeys had continued access to water during fasting. We placed animals on a 12‐h light/dark cycle and provided dietary enrichment once daily with food items not part of the regular diet (fresh produce, dried produce, dry item) and visual and audio enrichment (i.e., television, radio).

To obtain non‐sedated free catch urine samples, we placed a clean pan (without shavings) the afternoon prior to collection (2:00–3:00 p.m.) and aspirated approximately 10 mL of urine from the pan prior to 9:00 a.m. the following morning. We collected samples daily between day 2 and day 5, in addition to day 8, day 10 (females only), and day 11 (males only), for a total of six FC samples per monkey. Three of these were obtained prior to a sedated study and were therefore fasted samples, while the other three were non‐fasted. We centrifuged samples at 2100 *g* for 5 min at 4°C to isolate urine from solid particulates (food, stool).

We obtained sedated cystocentesis samples from each monkey on scheduled imaging days: day 2 (echocardiography), day 8 (pre‐MRI screening X‐ray), and day 10 (cardiac MRI) for females, and day 5 (echocardiography), day 8 (pre‐MRI screening X‐ray), and day 11 (cardiac MRI) for males. We collected a total of three cystocentesis samples per monkey that were matched with three of the six FC samples. On imaging/cystocentesis days, we sedated monkeys with intramuscular ketamine (15 mg/kg) and transported them to the respective imaging/procedure area where we intubated and anesthetized them with 1%–2% inhaled isoflurane gas. We performed cystocentesis on all animals within 15 min of starting isoflurane. We placed monkeys in a supine recumbent position and cleaned the skin of the lower abdomen superior to the pelvic bone with chlorhexidine solution. We then inserted a 1.5‐in. 22‐gauge needle on a 10‐mL syringe into the bladder under ultrasound guidance and aspirated up to 10 mL of urine.

Urine samples were mixed well before aliquoting. For angiotensin peptide analysis, we immediately combined 5 mL of urine with 100 μL of 6 N HCl in a 15 mL polypropylene vial on ice. Separate urine aliquots without preservative were prepared for alpha‐klotho, creatinine, ACE, ACE2, and aldosterone. Alpha‐klotho aliquots of 300 μL were frozen immediately after collection on dry ice, and all urine aliquots were stored at −80°C until analysis.

### 2.3 Urinary Biomarker Analysis

The Biomarker Analytical Core at WFUSM measured the concentrations of urine creatinine as well as RAAS related biomarkers including ACE, ACE2, Ang‐(1–7), AngII, aldosterone, and alpha‐klotho according to established protocols, as detailed below.

Urinary angiotensin peptides [Ang‐(1–7), Ang II] were isolated by solid‐phase extraction using Sep‐Pak cartridges according to established plasma Sep‐Pak protocols modified for urine samples [26, 27]. Angiotensin II was quantified by radioimmunoassay using an IBL‐America/DIA source Angiotensin II RIA kit (Cat Number RB320RUO). Angiotensin‐(1–7) was quantified using a radioimmunoassay with radio‐iodinated tracer and Angiotensin‐(1–7) standards [28, 29]. Assay sensitivities of the Ang II and Ang‐(1–7) RIAs were 2.0 and 2.5 pg/mL, respectively. The peptide content was corrected for recovery by addition of a trace amount of 125I‐Ang II prior to extraction. Angiotensin peptide concentrations were corrected for extraction recovery, as previously described [26, 27]. The intra‐ and inter‐assay coefficient of variation (CV) for Ang II is 12% and 22%, respectively, while that for Ang‐(1–7) is 8% and 20%.

Urinary aldosterone was measured using the same Tecan/IBL‐International aldosterone radioimmunoassay protocol used for serum aldosterone (Cat Number MG13051), with an additional urine hydrolysis step before analysis. 100 μL of urine was incubated with 1 mL of 0.1 M hydrochloric acid for 15–20 h at 30°C. A 50‐μL aliquot of the hydrolyzed sample was then assayed with 150 μL of zero calibrator, and the result was corrected for recoveries. Manufacturer reported assay sensitivity is 27.74 pmol/L, and intra‐ and inter‐assay CV is 5% and 6.6%, respectively [27].

Urinary alpha‐klotho was measured using a human soluble alpha‐klotho sandwich ELISA kit (IBL‐America/Immuno‐Biological Laboratories, Cat. 27998), as previously described [30]. Manufacturer‐reported intra‐ and inter‐assay CVs ranged from 2.7%–3.5% and 2.9%–11.4%, respectively, depending on sample concentration. Sensitivity is 6.15 pg/mL. Urinary ACE and ACE2 protein concentrations were measured using human sandwich ELISA kits from RayBiotech (ACE: Cat. ELH‐ACE; ACE2: Cat. ELH‐ACE2) with detection levels of 83 and 25 pg/mL, respectively. For both ACE and ACE2, intra‐assay CV is < 10% and inter‐assay CV is < 12%. Urinary creatinine was measured using a colorimetric urine creatinine assay, with intra‐assay CV 1.1%–2.1% and inter‐assay CV 1.6%–1.9% (Metra, Cat. 8009).

Raw urinary biomarker concentrations were reported as pg/mL for Ang‐(1–7), Ang II, and alpha‐klotho; ng/mL for ACE, ACE2, and aldosterone; and mg/dL for creatinine. Values below the minimum detectable concentration were reported as less than the assay detection limit (3 cystocentesis creatinine values, 1 FC and 3 cystocentesis ACE2 values, 1 cystocentesis Ang II value, 4 FC and 1 cystocentesis alpha‐klotho value). We included these values below the minimum detectable concentration in our analyses by substituting the lower detection limit divided by √2. This is a widely established approach for handling concentrations below analytical detection limits [31]. Values above the reportable range were repeated with dilution (8 FC Ang II values).

We had several missing or excluded analytes due to collection or analysis limitations:

- One FC ACE2 timepoint from a single animal was above the upper limit of detection and unable to be diluted for repeat analysis. This vervet maintained all three FC‐cystocentesis pairs but only had five total FC samples for ACE2.
- One cystocentesis timepoint from a single animal did not have adequate volume collected to measure Ang‐(1–7) or AngII. This vervet maintained all six FC samples for these analytes, but only had two cystocentesis samples and FC‐cystocentesis pairs for Ang‐(1–7) and AngII.
- One FC urine sample from a single animal was excluded entirely due to significant contamination/dilution from drinking water into the collection pan. This vervet maintained all three cystocentesis samples, but only had five FC and two FC‐cystocentesis pairs for all analytes.

We normalized all RAAS analyte concentrations to urine creatinine in accordance with conventional approaches to account for differences in urinary concentration [32, 33, 34, 35, 36]. Because urine creatinine can also vary by collection condition, we also compared the variation in raw creatinine concentrations between groups.

### 2.4 Statistical Analysis

We performed all statistical analyses in R using the lme4, lmerTest, and emmeans packages. For each analyte, we evaluated day‐to‐day variability separately for FC (six FC samples per monkey) and cystocentesis (three cystocentesis samples per monkey) urine samples. We calculated CVs across repeated samples within each monkey and summarized this as the mean ± standard deviation for each analyte. CVs were classified as low (≤ 10%), intermediate (10%–20%), high (20%–30%), or very high (> 30%) [37].

We compared the median CV in FC and cystocentesis urine samples for each subject for each biomarker using Wilcoxon matched‐pair tests with *p*‐values adjusted for multiple comparison using the false discovery rate (FDR) method. Statistical significance was defined as FDR‐adjusted *p* value < 0.05.

In exploratory analyses, we compared median CVs calculated from fasted FC samples with CVs from non‐fasted FC samples and cystocentesis using Wilcoxon matched‐pair tests with FDR correction. We also compared median concentrations in fasted and non‐fasted FC samples using this same methodology.

To evaluate whether urinary RAAS concentrations changed following sedation, we compared paired FC and cystocentesis samples collected from the same monkeys on the same sampling dates using linear mixed‐effects models. Monkeys contributed three paired FC and cystocentesis samples, with exceptions to this noted above. Sedation (non‐sedated FC vs. sedated cystocentesis) was included as a fixed effect, while monkey ID and sampling date were included as random intercepts to account for repeated measurements and within‐subject pairing. Estimated marginal means were used to compare collection methods. *p*‐values were adjusted for multiple comparisons using the FDR method. Statistical significance was defined as FDR‐adjusted *p* value < 0.05.

Artificial intelligence (Claude) was used to identify and correct coding errors in R during statistical analysis.

## 3 Results

### 3.1 Day‐To‐Day Variability in Non‐Sedated Free‐Catch Urine and Sedated Cystocentesis Urine

We observed substantial day‐to‐day variability across all urinary RAAS biomarkers (Figures 2 and 3, Table S1). In FC urine samples, mean CVs ranged from 34.73% (standard deviation 14.75%) for aldosterone to 103.03% for alpha‐klotho (standard deviation 19.82%). All seven analytes fell within the very high CV category (> 30%). In cystocentesis urine samples, mean CVs ranged from 28.17% (standard deviation 18.28%) for ACE to 74.85% (standard deviation 32.6%) for alpha‐klotho. ACE fell within the high CV category (20%–30%), and the remaining six analytes within the very high category.

**FIGURE 2:**
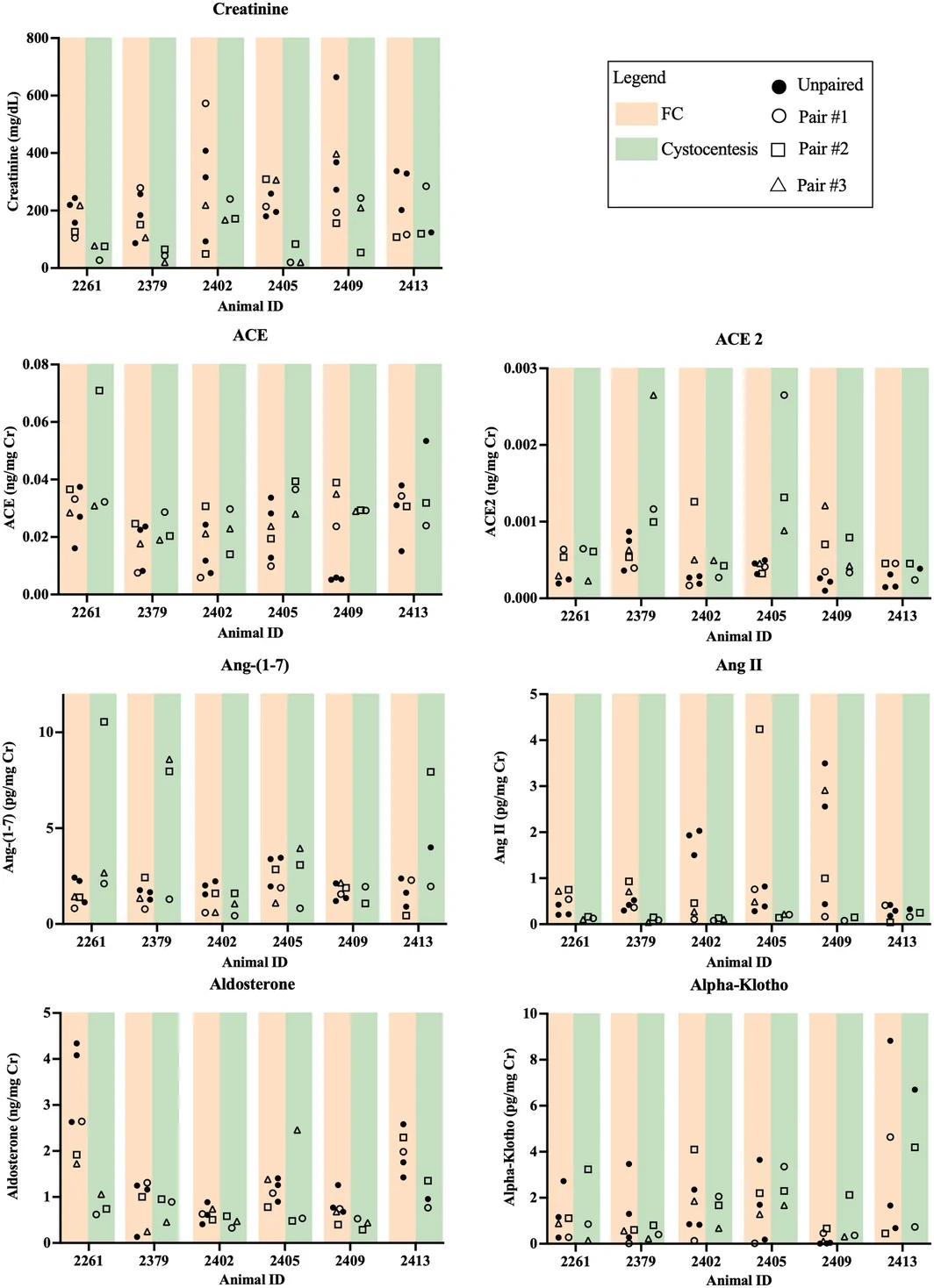
RAAS pathway urine concentrations in non‐sedated free catch and sedated cystocentesis samples for all subjects. All analytes normalized to time‐ and collection‐method matched urine creatinine concentration. Note both daily variability and individual differences between non‐sedated and sedated values. ACE, angiotensin converting enzyme; Ang, angiotensin; FC, free catch. *X*‐axis shows individual animal ID, with points within the shaded orange collected via non‐sedated FC and points within the green collected via sedated cystocentesis. Samples collected on the same day (pre‐ and post‐sedation) are denoted according to the symbols provided in the legend.

**FIGURE 3:**
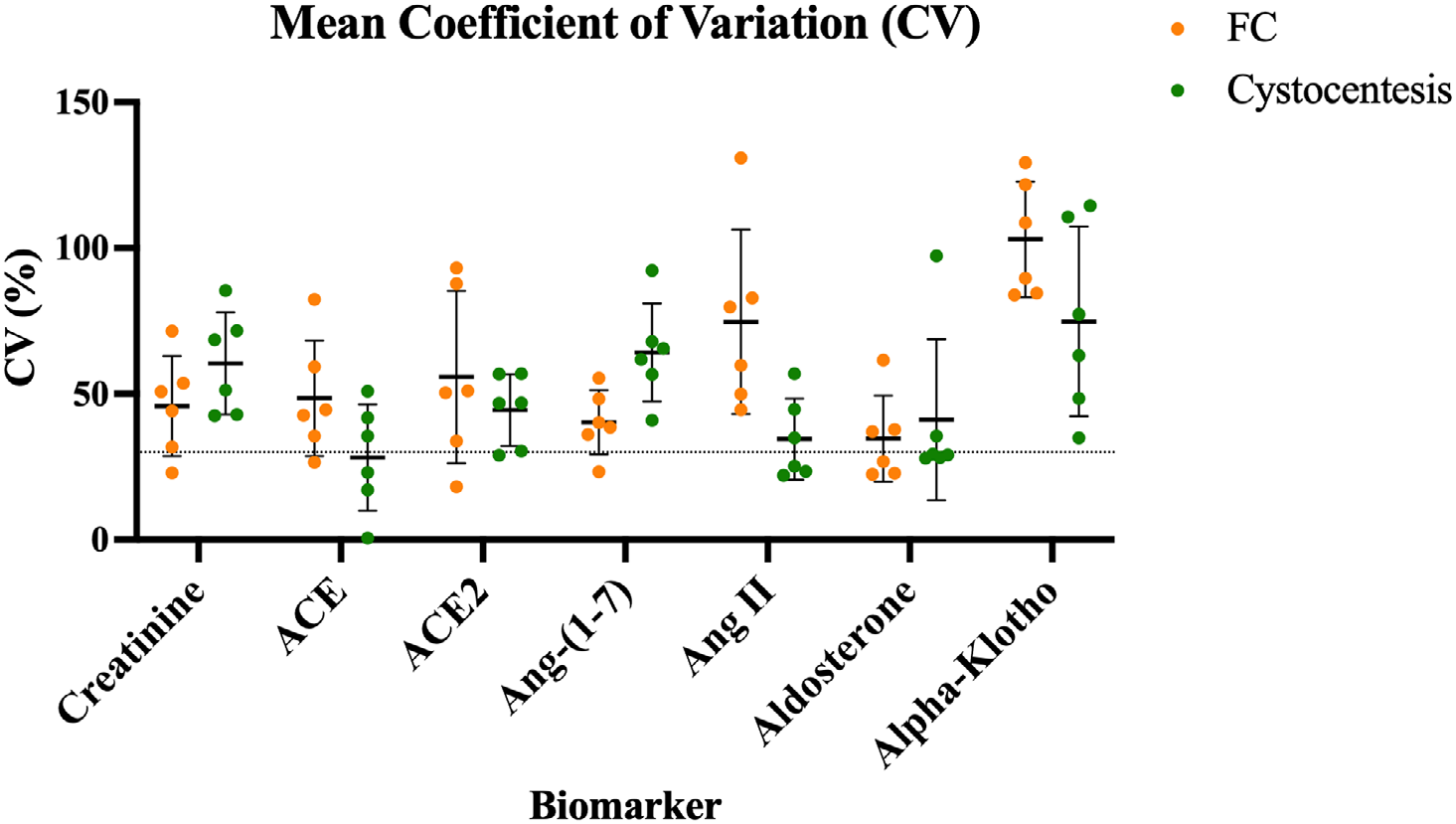
High variation in urinary RAAS analytes regardless of collection method. Individual data points represent CV for each subject from repeated sample collections for each measured component of the RAAS pathway, with mean CV indicated by the black line. Mean within‐subject CVs were > 20% for all analytes under both conditions. All values above the dashed line (at 30%) represent those biomarkers with very high variability. ACE, angiotensin converting enzyme; Ang, angiotensin; FC, free catch.

We found that the collection method did not significantly impact CV for any biomarker after adjustment for multiple comparisons (Table S2). Ang‐(1–7) demonstrated the lowest unadjusted *p* value (median FC—cystocentesis difference = −24.305, unadjusted *p* value = 0.031), with all subjects demonstrating lower daily variability in FC samples compared to cystocentesis samples; however, this difference was not significant after FDR correction (FDR adjusted *p* value = 0.230). No significant differences were observed in daily variability between collection methods for creatinine, ACE, ACE2, Ang II, aldosterone, or alpha‐klotho.

In exploratory analyses of fasting state, mean CVs calculated from fasted FC samples remained high to very high and did not differ from non‐fasted FC CVs (all FDR‐adjusted *p* values ≥ 0.36) or fasted cystocentesis CVs (all FDR‐adjusted *p* values ≥ 0.77) (data not shown).

### 3.2 Differences in Urinary RAAS Biomarkers Between Non‐Sedated Free Catch and Sedated Cystocentesis Samples

Several biomarkers differed between paired non‐sedated FC and sedated cystocentesis samples (Figure 4, Table S3). Creatinine was significantly higher in FC samples compared with cystocentesis samples (mean FC − cystocentesis difference: 100.13 mg/dL, FDR‐adjusted *p* value = 0.044). Sedation differences were also seen with several creatinine‐normalized RAAS biomarkers. Ang‐(1–7) was significantly lower in non‐sedated FC samples compared with sedated cystocentesis samples (−2.126, FDR‐adjusted *p* value = 0.044), whereas Ang II and aldosterone were significantly higher in non‐sedated FC samples (Ang II: 0.611, FDR‐adjusted *p* value = 0.044; aldosterone: 0.418, FDR‐adjusted *p* value = 0.044). ACE, ACE2, and alpha‐klotho did not differ significantly between collection methods after FDR correction. In exploratory analyses of fasting state, concentrations did not differ between fasted and non‐fasted FC samples for any analyte (all FDR‐adjusted *p* values ≥ 0.51) (data not shown).

**FIGURE 4:**
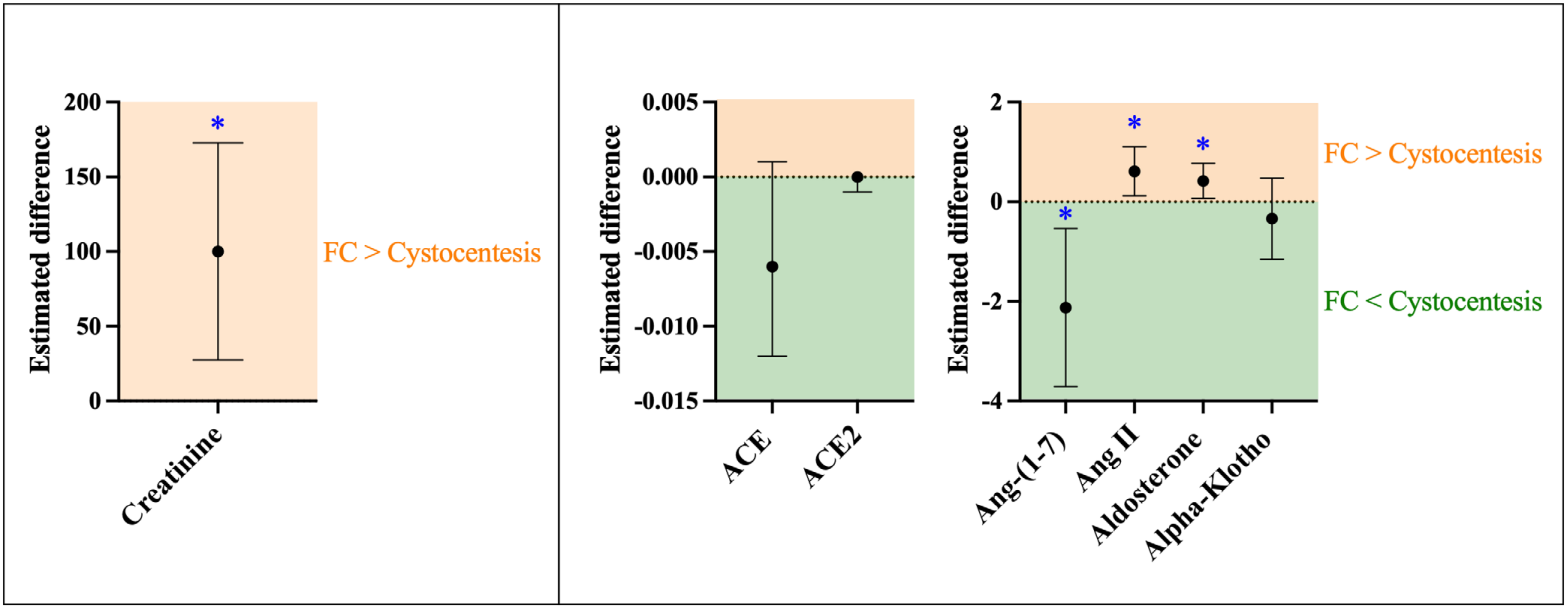
Estimated differences in biomarkers between FC and cystocentesis urine samples. Data points represent the estimated difference in biomarker concentration between FC and cystocentesis samples with 95% confidence intervals. Significant differences after FDR correction denoted with blue asterisk (*p* < 0.05). The dotted line at *y* = 0 represents the point at which there is no difference in biomarker concentration in non‐sedated FC compared to sedated cystocentesis samples. All points above the dotted line (within the shaded orange) represent biomarkers that had higher concentrations in non‐sedated FC samples, while points below the dotted line (within the shaded green) represent biomarkers that had higher concentrations in sedated cystocentesis samples. ACE, angiotensin converting enzyme; Ang, angiotensin; FC, free catch. Units for each biomarker are as follows: Creatinine (mg/dL); ACE, ACE2, aldosterone (ng/mg Cr); Ang‐(1–7), Ang II, Alpha‐Klotho (pg/mg Cr).

## 4 Discussion

RAAS dysregulation has been repeatedly implicated in the pathogenesis of cardiovascular disease [4, 17, 38, 39]. However, validated and reproducible assays to adequately evaluate underlying disease mechanisms in animal models are lacking. NHP, such as the vervet monkey, offer a uniquely translational model to study spontaneous hypertension and developmental RAAS programming [8, 40, 41]. Measurement of urinary RAAS biomarkers may be particularly useful given rapid physiologic fluctuations, preanalytical factors, and sedation‐related effects on circulating biomarkers [42]. In this study, we characterized day‐to‐day variability of urinary RAAS biomarkers in vervet monkeys and evaluated whether concentrations differ between non‐sedated FC and sedated cystocentesis urine collection.

Our results indicate that most urinary RAAS biomarkers exhibit high to very high day‐to‐day variability in both FC and cystocentesis samples, and sedation influences concentration in an analyte‐specific manner.

### 4.1 Urinary RAAS Biomarkers Reflect Multiple Physiologic Compartments

Urinary biomarkers may provide insight into both systemic and intrarenal RAAS activity while circumventing some of the preanalytical challenges inherent to serum and plasma measurement. The intrarenal RAAS is recognized as a locally regulated system with distinct physiological characteristics from the systemic RAAS, including local synthesis of RAAS components and differing regulation mechanisms [38, 39]. While systemic RAAS contributes to cardiovascular disease through cardiac remodeling, vascular changes, sodium/water retention, and sympathetic activation, evidence suggests that the intrarenal RAAS independently contributes to cardiovascular disease through enhanced sodium retention and renal fibrosis [39]. The intrarenal RAAS is not fully independent of the systemic circulation; liver‐derived angiotensinogen is the primary source of renal angiotensinogen protein and angiotensin II, and much of proximal tubular renin is retrieved from the ultrafiltrate rather than synthesized locally. However, the intrarenal RAAS remains incompletely understood and difficult to disentangle from systemic and other tissue‐specific RAAS activity, as the relative contribution of each system may vary by disease state and patient factors [38, 39, 43].

Furthermore, the interpretation of urinary biomarkers is complex because multiple sources, including glomerular filtration of systemic components, tubular reabsorption, and local intrarenal synthesis, contribute to urinary analyte concentrations [44]. In healthy kidneys, urinary ACE, ACE2, Ang‐(1–7), and Ang II are thought to originate predominantly from intrarenal sources, while urinary aldosterone is thought to originate from systemic sources. However, the relative contributions of local synthesis versus filtered systemic components remain incompletely defined [44, 45]. Urinary alpha‐klotho, a negative regulator of the intrarenal RAAS, most likely reflects intrarenal expression independent of circulating levels [30]. However, this marker participates in multiple pathways and has not been validated as a specific surrogate measure of intrarenal RAAS activity. Because each analyte reflects a different mixture of systemic and intrarenal contributions, the reproducibility patterns described below should be interpreted with this underlying physiology in mind.

### 4.2 Urinary RAAS Daily Variability

RAAS biomarkers had very high CVs in non‐sedated FC urine, indicating substantial intraindividual day‐to‐day variability. Ang II and alpha‐klotho were particularly variable, with mean CVs of 74.66% and 103.03%, respectively. Both analytes are known to be unstable in unprocessed urine, and these findings align with other studies that have found significant analytical limitations when quantifying urinary angiotensin peptides and alpha‐klotho [8, 46].

Similarly, all sedated cystocentesis samples demonstrated high to very high CVs. When comparing the variability between non‐sedated FC and sedated cystocentesis, we anticipated that CVs would be lower from cystocentesis samples given the standardized collection and immediate processing with less time for analyte degradation, sample contamination, or evaporation. While ACE, ACE2, Ang II, and alpha‐klotho demonstrated lower mean CVs in sedated cystocentesis samples than the corresponding non‐sedated FC samples, Ang‐(1–7) and aldosterone showed higher mean CVs in sedated cystocentesis urine. However, when CVs were compared using Wilcoxon tests and adjusted for multiple comparisons, there were no significant differences. Although we would anticipate that both angiotensin [Ang‐(1–7) and Ang II] peptides would be vulnerable to degradation and therefore show similar trends [8], Ang‐(1–7) may be particularly unstable regardless of immediate processing with cystocentesis and may also be more sensitive to the hemodynamic effects of sedation discussed below.

The high day‐to‐day variability observed in this study is broadly consistent with existing data showing substantial intraindividual fluctuation in RAAS components [8, 9, 16, 47, 48]. For example, one study of hypertensive patients found CVs for plasma aldosterone, renin, and aldosterone‐to‐renin ratio ranging from 24% to 42% [9]. Urine aldosterone measurement may be used for confirmatory testing of primary hyperaldosteronism in humans; however, this is not used as a standalone screening tool or replacement for blood‐based screening due to high variability [49, 50].

To our knowledge, no studies have systematically evaluated the variability and reliability of urinary RAAS biomarkers in nonhuman primates. These findings suggest that single measurements may inadequately characterize urinary RAAS activity and support the use of repeated sampling when feasible.

### 4.3 Effects of Sedation and Collection Method on Urinary RAAS

Given that studies in NHP models are often done under sedation or anesthesia, we examined whether paired measurements before and after sedation would be comparable. Sedating and anesthetic medications are expected to have agent‐specific effects on RAAS mediated through sympathetic nervous system modulation and hemodynamic physiologic changes. Therefore, samples obtained during sedation may not reflect baseline RAAS activity [11, 12, 18, 19, 20, 21, 22, 23, 24].

Ketamine, used to sedate vervets for capture in the present study, has complex effects on RAAS activity and existing evidence is limited. In one study of 10 human subjects, ketamine injection was associated with increased blood pressure and heart rate without rapid changes in plasma renin values [11]. Meanwhile, in another small human study, ketamine injection was associated with increased blood pressure, as well as a rapid decrease in Ang II and increase in renin concentration and activity [12]. Effects are also context‐dependent: in sodium‐repleted rats, ketamine did not increase plasma renin activity, but all anesthetic agents tested (including ketamine and inhaled anesthetics) increased plasma renin activity in sodium‐depleted rats [20, 21].

Inhaled anesthetics, such as isoflurane used in our study, are associated with increased plasma renin activity and plasma aldosterone concentrations. For example, in surgical patients receiving isoflurane, plasma renin activity rose significantly before surgical stimulation, and plasma aldosterone concentrations reached approximately three times baseline values during surgery [19].

Importantly, nearly all data on anesthetic‐RAAS interactions derive from rodent models and human surgical patients. In the present study, ketamine was administered at the time of capture, while isoflurane was administered for up to 15 min prior to urine collection. Based on this, we expected sedated cystocentesis samples to show signs of renin release [i.e., increased ACE, ACE2, Ang II; decreased Ang‐(1–7)] and increased aldosterone [6, 7, 12, 42]. However, we do not see physiologically consistent differences, and the differences we do see are not in the anticipated direction. Ang‐(1–7) is higher in sedated urine samples while Ang II and aldosterone are lower in sedated urine samples. ACE, ACE2, and alpha‐klotho did not differ significantly between methods. These seemingly contradictory findings may represent negative feedback in RAAS, with a rapid increase in renin and downstream Ang II and aldosterone immediately following ketamine sedation, which ultimately exerts negative feedback on the cascade, decreasing renin, Ang II, and aldosterone and increasing counterregulatory components such as Ang‐(1–7) by the time of sample collection.

Overall, sedation appears to have complex, analyte‐specific effects on urinary RAAS biomarkers that do not follow the expected pattern based on the known hemodynamic impacts of these medications. This likely reflects a combination of the self‐regulatory nature of RAAS and high baseline variability. To our knowledge, no studies have directly examined the effects of sedation or anesthesia on urinary RAAS biomarker concentrations in any species, and we would urge caution in the interpretation of urinary RAAS analytes in the future.

### 4.4 Limitations

Several limitations should be acknowledged. First, the sample size of only six vervets limited statistical power to detect modest differences between collection methods, particularly given the demonstrated variation. In addition, the number of repeated samples differed between collection methods (6 FC vs. 3 cystocentesis), which may have influenced the quality of variability estimates. Animals were also fasted for the matched FC and cystocentesis samples but not the remaining FC samples, so FC variability estimates combine fasted and non‐fasted conditions. Fasting may alter hydration, electrolyte balance, and urine concentration. Exploratory analyses found no significant differences between fasted and non‐fasted states; however, small sample sizes limit power to detect differences. Missing data, particularly in the two subjects who had two paired FC‐cystocentesis for some analytes rather than 3, may further decrease the quality of estimates. Sampling intervals were also unequal: FC samples were collected daily for 4 days followed by intervals of 2–3 days, and cystocentesis samples were separated by 2–6 days depending on sex. Because RAAS biomarkers fluctuate over time, uneven intervals may have affected variability estimates. Further, because the study included only healthy adult vervets, the magnitude and pattern of variability may differ in animals with hypertension, renal disease, or altered RAAS activity. Despite low sample size, the significant intra‐animal variability in all urinary RAAS analytes clearly demonstrates the need for large sample sizes and repeated collections in any study aiming to accurately characterize RAAS pathophysiology in a disease model.

Because sedation and collection method were inseparable in the present study, it is difficult to determine the relative effects of each variable. FC samples collected from cage pans may have been subject to evaporative concentration or environmental contamination, while cystocentesis samples may have been influenced by sedation‐related physiologic changes, as detailed above. Notably, urine creatinine concentrations were significantly higher in FC samples (FDR‐adjusted *p* value = 0.04). The higher creatinine in FC samples may reflect the more concentrated nature of pan urine collection, which accumulates over extended periods and is subject to evaporation [15, 51]. In contrast, cystocentesis samples represent a single time‐point aspiration from the bladder, where urine has had variable dwell time and may be diluted by ongoing ureteral inflow [52]. Differences in creatinine introduce further variability into the RAAS biomarkers, making interpretation more difficult. We evaluated alternative approaches, including variability in non‐normalized and albumin‐normalized biomarkers, which showed similarly very high variation, so we ultimately proceeded with creatinine normalization, the standard normalization strategy for urine biomarkers [32, 33, 34, 35, 36].

While we would have preferred a more controlled environment to account for factors, we were limited by staffing and logistics. For example, the timing of ketamine sedation to sample collection was imprecise due to animal transport and protocol logistics. These findings underscore that the collection method is a preanalytical variable that must be standardized within studies, and that creatinine normalization does not fully correct for collection‐method‐dependent differences in urine concentration.

## 5 Conclusions

Urinary RAAS biomarkers demonstrate substantial within‐subject variability in healthy vervet monkeys, indicating that single measurements inadequately reflect baseline RAAS activity. Future studies should take care to reduce sources of variability discussed here, including standardizing collection method and timing, sample processing, and anesthesia exposure, and should consider repeated sampling when feasible. Even with such precautions, urinary RAAS biomarkers should be interpreted with caution to avoid misclassification of biologic activity, and measurements obtained under different collection conditions should not be assumed to be interchangeable. Future work directly comparing urinary and circulating RAAS biomarkers and separating collection method from anesthesia effects is needed to further characterize sources of variability detailed here. These findings provide an important framework for the design and interpretation of future urinary biomarker studies in nonhuman primates.

## Funding

Research reported in this publication was supported by the National Heart, Lung, and Blood Institute of the National Institutes of Health under Award Number R01HL164434 (S.N.C.) as well as the Eunice Kennedy Shriver National Institute of Child Health & Human Development of the National Institutes of Health (The Wake Forest School of Medicine Training in Research Affecting Child Health (TRAC) Program) under Award Number R25HD111367 (M.E.G.). The Vervet Research Colony is supported as a biomedical resource by the Office of the Director of the National Institutes of Health under Award Number P40OD010965 (M. Jorgensen). The content is solely the responsibility of the authors and does not necessarily represent the official views of the National Institutes of Health.

## Ethics Statement

The authors confirm adherence to the ethical policies of the journal, as noted on the journal's author guidelines page. All animal studies were performed at Wake Forest University School of Medicine (WFUSM) and complied with international, national, and institutional guidelines for humane animal treatment, including those set forth by the US National Research Council “Guide for the Care and Use of Laboratory Animals” and the US Public Health Service “Policy on Humane Care and Use of Laboratory Animals” and “Guide for the Care and Use of Laboratory Animals.” All studies were conducted following approval of the WFUSM Institutional Animal Care and Use Committee (protocol number A24‐016).

## Conflicts of Interest

The authors declare no conflicts of interest.

## Supporting information


**Table S1:** Mean CV (± SD) of repeated measurements from six vervets for each RAAS analyte normalized to creatinine.

**Table S2:** Median FC—Cystocentesis difference in CV for each RAAS analyte normalized to creatinine.

**Table S3:** Estimated marginal means for paired FC versus cystocentesis samples.

## Data Availability

The data that support the findings of this study are available from the corresponding author upon reasonable request.
